# Molecular Phylogenetics and Mitochondrial Evolution

**DOI:** 10.3390/life12010004

**Published:** 2021-12-21

**Authors:** Andrea Luchetti, Federico Plazzi

**Affiliations:** Department of Biological, Geological and Environmental Sciences, University of Bologna, Via Selmi, 3, 40126 Bologna, BO, Italy; andrea.luchetti@unibo.it

The myth of a “typical” mitochondrial genome (mtDNA) is a rock-hard belief in the field of genetics, at least for the animal kingdom [1]. The first complete mitochondrial genomes were published in the 1980s [2,3,4,5]; since then, thousands of mtDNAs have been studied, and it is now well evident that only a few features (if any) are conserved among eukaryotic mtDNAs. Nonetheless, mtDNA has demonstrated, and it is everyday demonstrating, its suitability as a phylogenetic marker, ranging from population to phylum scale. In many cases, however, incompatibility issues arose between mitochondrial and nuclear phylogenies, and they have to be reconciled case by case.

The present Special Issue is an attempt to set and assess the state-of-art of mitochondrial genomics, with special reference to the suitability of mtDNAs for phylogenetic inference. The work by Formaggioni and colleagues [6] opens the Special Issue with a broad-scale analysis of the current knowledge on mitochondrial genomics, presenting the largest-to-date database of mtDNA features, which was made freely available to the scientific community. Similarly, the review by Parakatselaki and Ladoukakis [7], which closes the Special Issue, is specifically focused on mitochondrial heteroplasmy, a well-known phenomenon which is in fact neglected among the dogmatic “widespread” features of mitochondrial genomics, which include the strict maternal inheritance. Within this frame, several examples are provided of the usefulness of mtDNA for taxonomy, population genetics, and phylogenomics.

Furfaro and Mariottini [8,9] demonstrate the potential of mitochondrial rDNA secondary structures to unravel the taxonomy of a controversial gastropod family, Myrrhinidae, with the original description of a new genus; moreover, Dotsev and colleagues [10] address the taxonomic status of the Northernmost Snow Sheep (*Ovis nivicola*) using the well-exploited mitochondrial gene cytochrome *b*.

Several example of mtDNA-based population genetics are provided. Different strains of the cattle protozoan parasite *Theileria parva* are identified using mitochondrial single nucleotide polymorphisms in the work by Mwamuye and colleagues [11]; the population structure of the endangered freshwater mussel *Unio crassus* from Eastern Europe is unraveled by Kilikowska and colleagues [12] using one nuclear and two mitochondrial markers; similarly, the population genetics of the Portuguese oyster *Crassostrea angulata* is addressed by Chiesa and colleagues [13]. Conversely, Alarcón-Elbal and colleagues [14] provide a high-quality example of tracking the complete life cycle the water mite *Arrenurus (Micruracarus) novus* by means of morphology interwoven with the sequence of mitochondrial genes *cox1* and *cytb* at different life stages.

Mitochondrial phylogeny and comparison with nuclear data is the main purpose of the work by Zhao and colleagues [15] on two subfamilies of fig wasps, Epichrysomallinae and Sycophaginae, which are investigated using a large cluster of ortholog nuclear genes, as well as complete mitochondrial genomes; by Zadra and colleagues [16] on the genus *Aedes*, who found a strong and consistent incongruence between nuclear and mitochondrial phylogenetic inference (with special reference to dating), whose disentanglement is thoroughly discussed. Finally, Xia [17] reports on proper codon degeneration techniques to avoid phylogenetic artifacts when a mitochondrial phylogeny is inferred–a new method is implemented and applied to mammalian and avian lineages.

Furthermore, new complete mtDNAs are hereby reported. Arcila-Galvis and colleagues [18] present the complete mitochondrial genome of a plant fungal pathogen, *Pseudocercospora fijiensis* (Ascomycota: Pezizomycotina), as well as the phylogenetic reconstruction of the family Mycosphaerellaceae, providing clues for multiple invasions on introns within mitochondrial genes. Conversely, Johansen and colleagues [19] present the complete mitochondrial genome of a sea anemone, *Stichodactyla haddoni*, that shows group I introns harboring expressed open reading frames and supernumerary genes, and challenges current views of the Actiniidae taxonomy.

Concluding, the present Special Issue highlights the effectiveness of mitochondrial-based analyses in different fields of evolutionary biology, on one hand, while highlighting future, promising perspectives of research in the field of mitochondrial genetics, on the other hand, a field where unexpected is expected and the exception is the rule.
